# Coherence-Enhanced Single-Qubit Thermometry out of Equilibrium

**DOI:** 10.3390/e26070568

**Published:** 2024-06-30

**Authors:** Gonçalo Frazão, Marco Pezzutto, Yasser Omar, Emmanuel Zambrini Cruzeiro, Stefano Gherardini

**Affiliations:** 1 Instituto Superior Técnico, Universidade de Lisboa, 1049-001 Lisboa, Portugal; 2 Instituto de Telecomunicações, 1049-001 Lisboa, Portugal; 3 Physics of Information and Quantum Technologies Group, Centro de Física e Engenharia de Materiais Avançados (CeFEMA), 1049-001 Lisboa, Portugal; 4 PQI—Portuguese Quantum Institute, 1600-531 Lisboa, Portugal; 5 Istituto Nazionale di Ottica del Consiglio Nazionale delle Ricerche (CNR-INO), Largo Enrico Fermi 6, I-50125 Firenze, Italy; 6 European Laboratory for Non-Linear Spectroscopy, Università di Firenze, I-50019 Sesto Fiorentino, Italy

**Keywords:** quantum thermodynamics, quantumm thermometry, quantum fisher information, generalized amplitude damping channel, quantum coherence, quantum simulation with optics

## Abstract

The metrological limits of thermometry operated in nonequilibrium dynamical regimes are analyzed. We consider a finite-dimensional quantum system, employed as a quantum thermometer, in contact with a thermal bath inducing Markovian thermalization dynamics. The quantum thermometer is initialized in a generic quantum state, possibly including quantum coherence with respect to the Hamiltonian basis. We prove that the precision of the thermometer, quantified by the Quantum Fisher Information, is enhanced by the quantum coherence in its initial state. We analytically show this in the specific case of qubit thermometers for which the maximization of the Quantum Fisher Information occurs at a finite time during the transient thermalization dynamics. Such a finite-time precision enhancement can be better than the precision that is achieved asymptotically.

## 1. Introduction

Quantum thermometry aims at inferring the temperature of a thermal bath, or thermal reservoir, through the coupling with a quantum system [1]. In the quantum regime, any measurement device (thus, even a thermometer) is invasive to a given extent [2,3]. Hence, from an estimation perspective [4,5], the ultimate goal of thermometry is to determine the conditions under which the reconstruction of the temperature of a thermal bath can be effectively attained. Quantum metrology gives us the tools to achieve this task [6,7,8,9], in terms of the *Quantum Fisher Information* [10,11,12,13,14] applied to quantum thermometry [15,16,17]. In the case where only a few measurement records can be obtained, or no prior knowledge about the thermalization dynamics is available, global thermometry has recently been proposed [18]. The merit of such an approach is to identify in the mean logarithmic error an appropriate figure of merit for quantum thermometry. Moreover, it is worth recalling the framework of collision quantum thermometry [19,20,21] that identifies the possibility to probe the temperature of an environment by using correlated qubits within a sequential setting as it occurs in a collision model [22,23,24,25].

So far, some works have already analyzed how quantum thermometry can be employed in several quantum platforms for quantum technology. Among them, we would mention a three-level transmon circuit [26], a pair of trapped ions [27], a mechanical oscillator in the nonlinear regime [28], micromechanical resonators [29], Bose–Einstein condensates [30], ultracold atoms [31], cold Fermi gases [32], and even biological applications with cells [33,34].

In this paper, we set our analysis in the context of *qubit thermometers* [29,35,36], which have been experimentally tested in refs. [37,38] on a quantum optics platform. In particular, we assume that asymptotically (i.e., in the large time limit) the quantum thermometer is in the thermal state $\rho_{\beta }=e^{-\beta H}/Z_{\beta }$, with β denoting the inverse temperature of the bath, *H* the Hamiltonian of the thermometer and $Z_{\beta }$ the corresponding partition function. Then, as in [38], we consider that the thermometer weakly interacts with the thermal bath, so that the thermalization dynamics—to which the thermometer is subject—is well described by a Markovian master equation in Gorini–Kossakowski–Sudarshan–Lindblad (GKSL) form [39]. Because of the thermalization dynamics we study resulting in an asymptotic thermal state regardless of the initial state, the time-evolved state of the thermometer always encodes information about the temperature that we aim to infer.

In the conditions drawn above, in order to infer the temperature *T* of the thermal bath, one could wait for the full thermalization of the thermometer (i.e., wait for the state of the thermometer to be thermal), and then reconstruct *T* from its measure. However, the time required by the thermometer to thermalize can be very large, with the consequence that in a nanoscopic setting, other sources of error probably arise. This would have the effect of disturbing the state of the thermometer, spoiling the information on *T*.

In this paper, we follow a nonequilibrium approach for quantum thermometry [40] that relies on measuring the (time-dependent) state of the thermometer, while the thermalization is still active. From these measurements, the temperature of the bath is reconstructed. To guide this inference of the temperature with the best precision possible, we compute the Quantum Fisher Information, which depends on time, on the initial state of the thermometer (before it is put in contact with the thermal bath), and on the parameters of the GKSL master equation. The Quantum Fisher Information is a proper quantifier for also evaluating the precision of quantum thermometry. This is because for any unbiased estimator (in our case, the measurements of the quantum thermometer’s state), the uncertainty of the estimate (here, the reconstructed temperature) is bounded from below by the Quantum Fisher Information, according to the quantum Cramér-Rao bound [14,15,41,42]. Such a bound is tighter the larger the number of independent experiments performed to estimate the temperature.

Accordingly, the aim of this paper is to look for both the time in the transient thermalization dynamicsand the initial state of the thermometer, such that the Quantum Fisher Information is maximized. The result of this optimization is expected to guide experimentalists to set the optimal conditions, allowing them to carry out the thermometry task with high precision. In this regard, notice that the computation of the Quantum Fisher Information has to be performed before the thermometry experiments, requiring some a priori knowledge of the thermalization dynamics. For example, in the setting implemented experimentally in [38], which we consider in the following, one implicitly assumes that the thermalization dynamics of the quantum thermometer are well described by a master equation in the Markovian regime. We conclude the paper by discussing a possible experimental test of our results on the quantum optics platform in ref. [38], and we provide some outlook for possible future works.

## 2. Nonequilibrium Quantum Thermometer

In this section, we introduce the model of a thermometer as a *N*-level quantum system, interacting with a thermal bath in the weak-coupling regime. Here, we build upon known results in open system dynamics following references cited in Section 1 by introducing a Markovian master equation for a finite-level quantum system undergoing a thermalization process. Such a quantum master equation takes as input the reduced density matrix of the system. After the general treatment of an *N*-level quantum system, we focus on a qubit thermometer (N=2) for which we give most of the analytical results in this work.

The following assumptions are made: (1) The initial state of the thermometer and bath are uncorrelated; (2) The action of the thermometer on the bath is negligible, so the bath always remains in a thermal state; (3) The rotating wave approximation is valid: fast oscillating terms in the thermometer–bath dynamics, when compared with the thermometer time scale, are neglected. As a result, after tracing out the environment degrees of freedom, the dynamics of the quantum thermometer are governed by a Lindblad master equation [39,43]:

(1) $$\dot{\rho }\left(t\right)=-i\left[H,\rho (t)\right]+\sum_{i,j=1;\;\;i\neq j}^{N}\left(L_{ij}\rho \left(t\right)L_{ij}^{†}-\frac{1}{2}\left\{L_{ij}^{†}L_{ij},\rho \left(t\right)\right\}\right),$$

where *ℏ* is set to 1, and the Hamiltonian is

(2) $$H=\sum_{j=1}^{N}\epsilon_{j}|\epsilon_{j}\rangle \;\langle \epsilon_{j}|,$$

with eigenvalues ${\left\{\epsilon_{j}\right\}}_{j=1}^{N}$ arranged in order of increasing energy, and eigenvectors ${\left\{|\epsilon_{j}\right\}}_{j=1}^{N}$. In Equation (1), the thermalization dynamics induced on the thermometer by the interaction with the thermal bath is described via the jump operators $L_{ij}\equiv \sqrt{\Gamma_{ij}}|\epsilon_{i}\rangle \;\langle \epsilon_{j}|$ [43,44,45]. The transition rates $\Gamma_{ij}$ from state *j* to state *i* are given by

(3) $$\begin{array}{c} \Gamma_{ij}=\left\{\begin{array}{cc} \gamma (n_{ij}+1) & \;\mathrm{for}\;i<j\\ 0 & \;\mathrm{for}\;i=j\\ \gamma \;n_{ji} & \;\mathrm{for}\;i>j \end{array}\right. \end{array}$$

with γ>0 having dimension of [time]^−1^, and $n_{ij}$ denoting the thermal ratios

(4) $$\begin{array}{c} n_{ij}\equiv n_{ij}\left(\beta \right)=\frac{1}{e^{\beta \omega_{ij}}-1}\;, \end{array}$$

where $\omega_{ij}\equiv \epsilon_{j}-\epsilon_{i}$ and $\omega_{ij}>0$ for i<j. Since Equation (1) is a differential equation depending linearly on ρ, we can rewrite it as a system of linear differential equations for the terms $\rho_{ij}\equiv \langle \epsilon_{i}\left|\rho \right|\epsilon_{j}\rangle$ representing the projections of ρ on the energy eigenbasis:

(5) $$\begin{array}{cc} {\dot{\rho }}_{ii} & =\sum_{k=1}^{N}\left(\Gamma_{ik}\rho_{kk}-\Gamma_{ki}\rho_{ii}\right)\;, \end{array}$$

(6) $$\begin{array}{cc} {\dot{\rho }}_{ij} & =\left[-\frac{1}{2}\sum_{k=1}^{N}\left(\Gamma_{ki}+\Gamma_{kj}\right)+i\;\omega_{ij}\right]\rho_{ij}\;\mathrm{for}\;i\neq j\;. \end{array}$$

The time evolution of the diagonal population terms is decoupled from the evolution of the off-diagonal coherence ones. Hence, we address the dynamics of each of them separately. We stress that this is due to the specific thermalization dynamics we considered, i.e., the master Equation (1) with the jump operators $L_{ij}\equiv \sqrt{\Gamma_{ij}}|\epsilon_{i}\rangle \;\langle \epsilon_{j}|$.

### 2.1. Diagonal Population Terms

Let us denote the vector with the population terms as $p_{d}\equiv {\left[\begin{array}{c} \rho_{ii} \end{array}\right]}_{i}$. Equation (5) forms a linear differential system of *N* equations:

(7) $$\begin{array}{c} {\dot{p}}_{d}=A_{\beta }\;p_{d}, \end{array}$$

where $A_{\beta }\equiv {\left[a_{ij}\right]}_{ij}$ is the N×N transition matrix whose entries are given by

(8) $$\begin{array}{cc} a_{ij} & =\Gamma_{ij}\;, \end{array}$$

(9) $$\begin{array}{cc} a_{ii} & =-\sum_{k=1}^{N}\Gamma_{ki}\;. \end{array}$$

Out of these *N* differential equations, only N−1 are linearly independent, since $A_{\beta }$ has N−1 non-null eigenvalues, as demonstrated in Appendix A. Notice that Equation (9) is a direct consequence of probability conservation in terms of the normalization of ρ for any time: $\sum_{i}\rho_{ii}=1$. Moreover, the dependence of the matrix $A_{\beta }$ on β is made explicit through Equations (3) and (4). Hence, in solving the differential system (7), one obtains

(10) $$\begin{array}{c} p_{d}\left(t,\beta \right)\equiv e^{A_{\beta }t}p_{d}\left(0\right). \end{array}$$

As a consistency check, in Appendix A.3, we show that the matrix $A_{\beta }$ has a single null eigenvalue, while the remaining N−1 eigenvalues are strictly negative. In fact, by direct substitution, the vector $\pi \left(\beta \right)\equiv {\left[\begin{array}{c} e^{-\beta \epsilon_{k}}/Z \end{array}\right]}_{k}$ containing the thermal populations $\pi_{k}\equiv \pi_{k}\left(\beta \right)=e^{-\beta \epsilon_{k}}/Z_{\beta }$ with $Z_{\beta }\equiv \sum_{k}e^{-\beta \epsilon_{k}}$ is the eigenvector of $A_{\beta }$ associated with the null eigenvalue: $A_{\beta }\;\pi \left(\beta \right)=0$. This implies that, as t→∞, all the other N−1 eigenvectors of $A_{\beta }$ go to zero exponentially fast, so that the diagonal terms converge asymptotically to the thermal distribution.

### 2.2. Off-Diagonal Coherence Terms

In Equation (6), each of the pairs composed by ${\dot{\rho }}_{ij}$ and ${\dot{\rho }}_{ji}$ for i≠j consists of two complex conjugates and thus dependent differential equations. So, the system of equations [Equation (6)] is comprised of the N(N−1)/2 independent equations given by ${\dot{\rho }}_{ij}=\left(-c_{ij}+i\;\omega_{ij}\right)\rho_{ij}$, with $c_{ij}\equiv \frac{1}{2}\left(\sum_{k=1}^{N}\Gamma_{ki}+\Gamma_{kj}\right)>0$. Hence, given the initial quantum coherence term $\rho_{ij}\left(0\right)$, the time evolution of $\rho_{ij}$ is

(11) $$\begin{array}{cc} \rho_{ij}\left(t\right) & =e^{-c_{ij}t}e^{i\omega_{ij}t}\rho_{ij}\left(0\right)\;. \end{array}$$

Accordingly, the modulus of the off-diagonal terms, namely $|\rho_{ij}\left(t\right)|=e^{-c_{ij}t}\left|\rho_{ij}\left(0\right)\right|$, vanishes exponentially fast with decay rate $c_{ij}$. The dependence of $c_{ij}$ on β is evidenced through Equations (3) and (4). This implies that ρ(t) asymptotically converges to a diagonal state, which is thermal in this case study.

### 2.3. Qubit Thermometer

We now focus the analysis of the thermometer dynamics on the case where the thermometer is a 2-level quantum system, namely a qubit, meaning N=2. As shown in Appendix A.3, the transition matrix $A_{\beta }$ can be written in terms of the thermal distribution of the fixed point of the thermalization map. For a qubit, it can be determined that $A_{\beta }$ simplifies to

(12) $$A_{\beta }=\frac{\gamma }{\pi_{1}-\pi_{2}}\left[\begin{array}{cc} -\pi_{2} & \pi_{1}\\ \pi_{2} & -\pi_{1} \end{array}\right],$$

where we recall $\pi_{k}\equiv e^{-\beta \epsilon_{k}}/Z_{\beta }$ with $\sum_{k}\pi_{k}=1$ and $\epsilon_{i}\leq \epsilon_{j}$ for i<j. The eigensystem of $A_{\beta }$ is given by the pairs eigenvalue–eigenvector $\left\{\lambda_{0}=0;\;v_{0}=\pi ={\left[\pi_{k}\right]}_{k}\right\}$ and $\left\{\lambda =\gamma {\left(\pi_{2}-\pi_{1}\right)}^{-1}<0;\;v_{1}=\left[-1;1\right]\right\}$, where the dependence of $\pi_{1}$, $\pi_{2}$, and λ on β is omitted here for better readability. Notice that the notation [·;·] stands for column vector.

We introduce the operators $S\equiv [v_{0},v_{1}]$ and Λ≡diag(0,λ) from the spectral decomposition of $A_{\beta }$ such that $A_{\beta }=S\Lambda S^{-1}$. Thus, after exponentiation, one can determine that

(13) $$\begin{array}{cc} e^{A_{\beta }t} & =Se^{\Lambda t}S^{-1}=\left[\begin{array}{cc} \pi_{1} & -1\\ \pi_{2} & 1 \end{array}\right]\left[\begin{array}{cc} 1 & 0\\ 0 & e^{\lambda t} \end{array}\right]\left[\begin{array}{cc} 1 & 1\\ -\pi_{2} & \pi_{1} \end{array}\right]\\ & =\left[\begin{array}{cc} 1-\pi_{2}\left(1-e^{\lambda t}\right) & \left(1-\pi_{2}\right)\left(1-e^{\lambda t}\right)\\ \pi_{2}\left(1-e^{\lambda t}\right) & e^{\lambda t}+\pi_{2}\left(1-e^{\lambda t}\right) \end{array}\right]. \end{array}$$

Consequently, the diagonal elements p(t,β,a) of the qubit thermometer’s state at the generic time *t* are

(14) $$\begin{array}{cc} p(t,\beta ,a) & =e^{A_{\beta }t}p_{0}\left(a\right)=\left[\begin{array}{c} \pi_{1}-e^{\lambda t}\left(\pi_{1}-\left(1-a\right)\right)\\ \pi_{2}-e^{\lambda t}\left(\pi_{2}-a\right) \end{array}\right]\\ \; & =\left(1-e^{\lambda t}\right)\pi +e^{\lambda t}p_{0}\left(a\right)\;, \end{array}$$

where $p_{0}\left(a\right)\equiv \left[1-a;a\right]$ is the vector collecting the diagonal elements of the initial state (at time t=0) of the qubit thermometer.

On the other hand, the quantum coherence term $\rho_{12}\left(t,\beta \right)$ reads as

(15) $$\rho_{12}\left(t,\beta \right)=e^{\lambda t/2}e^{i\;\omega_{12}t}\rho_{12}\left(0\right),$$

where $\rho_{12}\left(0\right)$ is the value at t=0.

We also show the analytical expression of the derivative of the qubit thermometer’s state with respect to β (the derivation is in Appendix B.1):

(16) $$\begin{array}{cc} \partial_{\beta }\left(p(t,\beta ,a)\right) & =\left(1-\pi_{2}\right)\;\omega_{12}\;\delta \left(t,\beta ,a\right)\;v_{1}, \end{array}$$

(17) $$\begin{array}{cc} \partial_{\beta }\left(\rho_{12}\left(t,\beta \right)\right) & =\alpha \left(t,\beta \right)\;\rho_{12}\left(t,\beta \right), \end{array}$$

with

(18) $$\begin{array}{cc} \alpha (t,\beta ) & \equiv -\left(1-\pi_{2}\right)\pi_{2}\;\omega_{12}\lambda^{2}t \end{array}$$

(19) $$\begin{array}{cc} \delta (t,\beta ,a) & \equiv 1-e^{\lambda t}+2t\lambda^{2}e^{\lambda t}\left(\pi_{2}-a\right)\;. \end{array}$$

Notice that the derivation of Equations (16) and (17) is used to determine the analytical expression of the quantum Fisher information for qubit thermometers in Section 4. For the sake of clarity, in Appendix B.2, we also report a discussion on the difference between using the partial and total derivative with respect to β. This becomes relevant when the initial state of the thermometer, before it is put in contact with the thermal bath, is thermal at a given inverse temperature $\tilde{\beta }$ (not necessarily different from β).

## 3. Quantum Fisher Information

In this section, we summarize some key concepts from the area of quantum parameter estimation and quantum metrology. We introduce the Quantum Fisher Information (QFI) and discuss its fundamental role, in that it provides a means of quantifying the precision of a parameter estimation process by means of the Cramér-Rao bound. After this, we present some analytic results and considerations about the QFI for *N*-level quantum systems in three specific cases of interest, namely when the system is initialized in a thermal state (Section 3.1), a diagonal state (Section 3.2), and a generic state with quantum coherence (Section 3.3). These results lay the groundwork for the treatment of a qubit thermometer, which follows in Section 4. We emphasize that, while the main focus of this work is on finite-time quantum thermometry, the discussion and treatment of the QFI in itself are independent of the dynamical properties of the system in the object: the QFI depends directly on the state of the thermometer at any time, as well as on the measurements we perform.

From this section onward, we address the following questions: (1) How much information about the inverse temperature β of a thermal bath can be extracted from a quantum thermometer in the transient thermalization dynamics? (2) Does an initial state $\rho_{0}$ with quantum coherence yield more information about β than its diagonal counterpart $\rho_{d}$? For clarity, $\rho_{d}$ is a density operator with only diagonal elements that are defined over the eigenbasis spanned by $\left\{|\epsilon_{j}\rangle \right\}$ with j=1,…,N. Hence, the difference between ρ and $\rho_{d}$ is a Hermitian complex matrix χ with off-diagonal elements only, containing the quantum coherence of the initial state that makes the energy levels of the thermometer Hamiltonian interfering. Moreover, we also stress that the ultimate goal of this paper is to understand, with analytical arguments, what the precision (i.e., the metrological limit) in inferring β is during the transience of the thermalization dynamics to which the quantum thermometer is subject.

The information that a quantum state, described in the general case by the density operator ρ(t,β), has at any time *t* about the parameter β of the thermal bath is quantified by the Quantum Fisher Information [10,11,12,14]. The latter is formally defined as

(20) $$F\left(\rho_{\beta }\left(t\right),\beta \right)=\mathrm{Tr}\left[\rho_{\beta }\left(t\right)L_{\beta }^{2}\left(t\right)\right],$$

where $L_{\beta }\left(t\right)$ is the *Symmetric Logarithmic Derivative* (SLD), and we outlined the dependence on the inverse temperature β (when present) by means of a subscript in $\rho_{\beta }\left(t\right)$ and $L_{\beta }\left(t\right)$. The definition of the SLD is implicitly given by the *Lyapunov equation*

(21) $$\partial_{\beta }\left(\rho_{\beta }\left(t\right)\right)=\frac{1}{2}\left(L_{\beta }\left(t\right)\rho_{\beta }\left(t\right)+\rho_{\beta }\left(t\right)L_{\beta }\left(t\right)\right)\equiv \frac{1}{2}\left\{\rho_{\beta }\left(t\right),L_{\beta }\left(t\right)\right\},$$

where $\partial_{\beta }$ denotes the partial derivative with respect to β, and {·,·} is the anticommutator. Importantly, the definition of the QFI in Equation (20) is also equivalent to the relation

(22) $$F\left(\rho_{\beta }\left(t\right),\beta \right)=\mathrm{Tr}\left[\partial_{\beta }\left(\rho_{\beta }\left(t\right)\right)L_{\beta }\left(t\right)\right],$$

whose validity can be checked by substituting (21) in (22) and using the cyclic property of the trace. As given by the quantum Cramér-Rao inequality [14,15,41,42], the QFI identifies a lower bound for the uncertainty in estimating the unknown parameter (here, β), as a function of the number *M* of independent experiments or trials performed for such an estimation. Formally, this means that, by denoting with Var(β) the variance of the β-estimate, the quantum Cramér–Rao inequality reads as

(23) $$\mathrm{Var}\left(\beta \right)\geq \frac{1}{MF(\rho_{\beta }\left(t\right),\beta )}\;.$$

The quantum Cramér–Rao bound provides the ultimate precision limit allowed by quantum mechanics, as long as the employed estimator is unbiased. More specifically, we may take $\Delta \beta \geq {\left(\sqrt{F(\rho_{\beta }\left(t\right),\beta )}\right)}^{-1}$, with M=1, as a quantifier for the *precision* of the β-estimate. Nevertheless, even if the unbiasedness requirement is not fulfilled by the estimator, the maximization of the QFI leads to a suboptimal solution for reducing the estimation uncertainty. With this spirit, in this paper, we analyze the main conditions that entail the maximization of the QFI for any time of the Markovian thermalization dynamics to which the chosen quantum thermometer is subjected.

Now, we compute the QFI in three distinct scenarios with an increasing level of complexity. (1) First, we determine the QFI of a thermal state at an inverse temperature $\tilde{\beta }$. Notice that, from now on, we use $\tilde{\beta }$ whenever we need to denote an inverse temperature that is not related to one of the thermal baths we aim to infer. (2) Then, we derive the QFI of a diagonal state $\rho_{d}$ with respect to the basis of *H*. States of this kind are produced by the thermalization dynamics in (1) in the case the quantum thermometer is initialized in a diagonal state but not necessarily thermal. (3) We show the general properties of the QFI (about β) of a generic density operator that, compared with $\rho_{d}$, also contains quantum coherences. This calculation is needed when, at the beginning of the thermalization dynamics (1), the quantum thermometer is initialized in a generic state ρ(0).

As a remark, the analysis below about the QFI, albeit focusing on the specific parameter β (the inverse temperature of a thermal bath) and on the thermalization dynamics, can be applied in a more general context. In fact, the analysis works regardless of the quantum dynamics returning the state on which the QFI is computed, and the properties of the QFI we determine are valid in principle in any scenario for quantum parameter estimation.

### 3.1. QFI of a Thermal Quantum State

Let us consider a thermal state of the Hamiltonian *H* in (2) at inverse temperature $\tilde{\beta }$, i.e.,

(24) $$\rho_{\tilde{\beta }}=\frac{e^{-\tilde{\beta }H}}{Z_{\tilde{\beta }}}=\sum_{j=1}^{N}\pi_{j}\left(\tilde{\beta }\right)|\epsilon_{j}\rangle \;\langle \epsilon_{j}|\;,$$

with $Z_{\tilde{\beta }}\equiv \mathrm{Tr}\left[e^{-\tilde{\beta }H}\right]=\sum_{j=1}^{N}e^{-\tilde{\beta }\epsilon_{j}}$. In order to determine the expression of the QFI of the thermal state $\rho_{\tilde{\beta }}$, we have to compute both the derivative $\partial_{\tilde{\beta }}\left(\rho_{\tilde{\beta }}\left(t\right)\right)$ and the SLD $L_{\tilde{\beta }}$ for the case study under analysis. As proved in Appendix C, it holds that

(25) $$\partial_{\tilde{\beta }}\left(\rho_{\tilde{\beta }}\right)=\sum_{j=1}^{N}\partial_{\tilde{\beta }}\pi_{j}\left(\tilde{\beta }\right)|\epsilon_{j}\rangle \;\langle \epsilon_{j}|=\sum_{j=1}^{N}\left({\langle H\rangle }_{\rho_{\tilde{\beta }}}-\epsilon_{j}\right)\pi_{j}\left(\tilde{\beta }\right)|\epsilon_{j}\rangle \;\langle \epsilon_{j}|\;,$$

where ${\langle H\rangle }_{\rho_{\tilde{\beta }}}\equiv \mathrm{Tr}\left[\rho_{\tilde{\beta }}H\right]=\sum_{j=1}^{N}\epsilon_{j}\pi_{j}\left(\tilde{\beta }\right)$ is the expectation value of the Hamiltonian of the quantum thermometer with respect to the thermal state $\rho_{\tilde{\beta }}$. In this way, given the expressions of $\rho_{\tilde{\beta }}$ and $\partial_{\tilde{\beta }}\left(\rho_{\tilde{\beta }}\right)$, we obtain the SLD $L_{\tilde{\beta }}$ that is given by the following diagonal matrix:

(26) $$L_{\tilde{\beta }}=\sum_{j=1}^{N}\left({\langle H\rangle }_{\rho_{\tilde{\beta }}}-\epsilon_{j}\right)|\epsilon_{j}\rangle \;\langle \epsilon_{j}|\;.$$

The validity of Equation (26) can be directly verified by substituting (26) in the Lyapunov Equation (21). As a result, the QFI about the inverse temperature $\tilde{\beta }$ of the thermal state $\rho_{\tilde{\beta }}$ is

(27) $$F\left(\rho_{\tilde{\beta }},\tilde{\beta }\right)=\sum_{j=1}^{N}{\left({\langle H\rangle }_{\rho_{\tilde{\beta }}}-\epsilon_{j}\right)}^{2}\pi_{j}\left(\tilde{\beta }\right)\equiv \mathrm{Var}{\left(H\right)}_{\rho_{\tilde{\beta }}}\;,$$

where $\mathrm{Var}{\left(H\right)}_{\rho_{\tilde{\beta }}}$ is the (thermal) variance of *H* computed with respect to the thermal state $\rho_{\tilde{\beta }}$.

For qubits, the thermal expectation value ${\langle H\rangle }_{\rho_{\tilde{\beta }}}$ and variance $\mathrm{Var}{\left(H\right)}_{\rho_{\tilde{\beta }}}$, [here equal to the QFI $F(\rho_{\tilde{\beta }},\tilde{\beta })$], are equal to

(28) $$\begin{array}{cc} {\langle H\rangle }_{\rho_{\tilde{\beta }}} & =\epsilon_{1}\pi_{1}\left(\tilde{\beta }\right)+\epsilon_{2}\pi_{2}\left(\tilde{\beta }\right)=\epsilon_{1}+\omega_{12}\pi_{2}\left(\tilde{\beta }\right)=\epsilon_{2}-\omega_{12}\left(1-\pi_{2}\left(\tilde{\beta }\right)\right) \end{array}$$

(29) $$\begin{array}{cc} \mathrm{Var}{\left(H\right)}_{\rho_{\tilde{\beta }}} & =\omega_{12}^{2}\pi_{2}\left(\tilde{\beta }\right)\left(1-\pi_{2}\left(\tilde{\beta }\right)\right), \end{array}$$

where $\omega_{12}=\epsilon_{2}-\epsilon_{1}$ is the spectral gap of the qubit Hamiltonian.

Given that $F(\rho_{\tilde{\beta }},\tilde{\beta })$ quantifies the information contained in $\rho_{\tilde{\beta }}$ about the inverse temperature $\tilde{\beta }$, it is worth asking what is the Hamiltonian *H* that maximizes $F(\rho_{\tilde{\beta }},\tilde{\beta })$. This problem, for a thermal state, has already been studied in [15], where it is explicitly stated that determining the spectrum of *H* with the largest possible variance at thermal equilibrium directly entails the maximization of the precision to access a given temperature. It is also shown that the solution to this problem (i.e., the maximization of $\mathrm{Var}{\left(H\right)}_{\rho_{\tilde{\beta }}}$) is provided by taking the energy spectrum of an effective two-level quantum system with energies $E_{-}$ and $E_{+}$ associated, respectively, to $N_{-}$ and $N_{+}\equiv N-N_{-}$ times degenerate ground and excited states. In this way, $\mathrm{Var}{\left(H\right)}_{\rho_{\tilde{\beta }}}$ is maximized in the case the degeneracy of the excited state is the largest possible, which is obtained by setting $N_{-}=1$. These considerations, of course, hold independently on the estimation algorithm one employs to estimate $\tilde{\beta }$.

### 3.2. QFI with an Initial Diagonal State

Let us now provide the formal expression of the QFI about an inverse temperature β of a density operator $\rho_{\beta ,d}$ with only diagonal elements. If the initial quantum state of the thermometer is mixed with respect to the eigenbasis of *H*, then no quantum coherence in such a basis arises, with the result that the state of the thermometer remains mixed for any time *t* as given by Equation (10). Hence, the expression of QFI discussed in this subsection can be applied to the state of the quantum thermometer at any time *t* of its dynamics, provided the thermometer is initialized in a mixed quantum state. Let us also remark that, while the thermometer may be initialized in a quantum state which bears no dependency on β, such a dependency is expected to arise as a consequence of the thermalization dynamics.

Using Equation (20), the QFI of the generic mixed quantum state $\rho_{\beta ,d}$ is

(30) $$F\left(\rho_{\beta ,d},\beta \right)=\mathrm{Tr}\left[\partial_{\beta }\left(\rho_{\beta ,d}\right)L_{\beta ,d}\right]=\mathrm{Tr}\left[\rho_{\beta ,d}L_{\beta ,d}^{2}\right],$$

where $L_{\beta ,d}$ denotes the SLD for the case study of deriving the QFI of a mixed quantum state. The SLD $L_{d}$ is implicitly defined by the Lyapunov equation $\partial_{\beta }\left(\rho_{\beta ,d}\right)=\left\{\rho_{\beta ,d},L_{\beta ,d}\right\}/2$. At this point, it is worth observing that, $\rho_{\beta ,d}$ being provided by a matrix with only diagonal elements, the corresponding derivative $\partial_{\beta }\left(\rho_{\beta ,d}\right)$ with respect to β is also a diagonal matrix, as well as the SLD $L_{\beta ,d}$. Therefore,

(31) $$L_{\beta ,d}\;\rho_{\beta ,d}=\mathrm{diag}\left({\left\{L_{\beta ,d}\left(k\right)\rho_{\beta ,d}\left(k\right)\right\}}_{k=1}^{N}\right)=\partial_{\beta }\left(\rho_{\beta ,d}\right)\;,$$

where diag(·) denotes a diagonal matrix whose diagonal is the vector (·), and $L_{\beta ,d}\left(k\right)$, $\rho_{\beta ,d}\left(k\right)$ are the *k*-th elements on the diagonal of $L_{\beta ,d}$ and $\rho_{\beta ,d}$, respectively. From Equation (31), the following relation follows directly, providing the formal expression for the diagonal elements of $L_{\beta ,d}$, i.e.,

(32) $$L_{\beta ,d}\left(k\right)=\frac{\partial_{\beta }\left(\rho_{\beta ,d}\left(k\right)\right)}{\rho_{\beta ,d}\left(k\right)}\;,\;k=1,\ldots ,N\;.$$

As a result,

(33) $$F\left(\rho_{\beta ,d},\beta \right)=\sum_{k}\frac{{\left(\partial_{\beta }\left(\rho_{\beta ,d}\left(k\right)\right)\right)}^{2}}{\rho_{\beta ,d}\left(k\right)}\;.$$

Before moving forward, we stress that the analytic solution of the optimization problem returning the initial state that maximizes the QFI in the case of diagonal and generic density operators is postponed to the next section, Section 4, which deals with qubit thermometers.

### 3.3. QFI with a Generic Initial State

In this subsection, we address the following question for any quantum thermometer undergoing the dynamics in Section 2: Does a density operator $\rho_{\beta }$ with quantum coherence yield more information about the inverse temperature β of a thermal bath than its classical counter-part $\rho_{\beta ,d}$?

For this purpose, let us write the generic density operator $\rho_{\beta }$ as $\rho_{\beta }=\rho_{\beta ,d}+\rho_{\beta ,\mathrm{coh}}$, where $\rho_{\beta ,d}$ is the diagonal density operator introduced in Section 3.2, while $\rho_{\beta ,\mathrm{coh}}$ is a null-diagonal operator (namely a hollow matrix) with off-diagonal elements, representing quantum coherence. For the sake of a simpler notation, in this section, we dropped the dependency on both *t* and β; in the remainder of the main text, we use them again whenever needed. Moreover, we recall that the time evolution of the diagonal and off-diagonal elements of the density operator ρ(t) (the solution of the thermalization dynamics in Section 2) are decoupled from each other, as given, respectively, by Equations (5) and (6).

Now, we are in the position to write the QFI F(ρ,β) of the state of the quantum thermometer (at a given time *t* that we do not specify), as composed by the QFI $F(\rho_{d},\beta )$ associated to the diagonal elements of ρ(t), plus an additional non-negative term whose expression we provide. We make abundant use of the following proposition that can be easily proved by direct substitution:

**Proposition** **1.** *Let C be an n×n hollow matrix (i.e., its diagonal elements are equal to zero). Moreover, let D be an n×n diagonal matrix. Then, both CD and DC are hollow matrices.*

We also introduce *L* and $L_{\mathrm{coh}}$ as the SLD of ρ and $\rho_{\mathrm{coh}}$, respectively, both defined with respect to the inverse temperature β, so that $2\partial_{\beta }\left(\rho \right)=\left\{\rho ,L\right\}$ and

(34) $$\begin{array}{c} 2\partial_{\beta }\left(\rho_{\mathrm{coh}}\right)=\left\{\rho_{\mathrm{coh}},L_{\mathrm{coh}}\right\}\;. \end{array}$$

Moreover, we define $\tilde{L}\equiv L-L_{d}$, where $L_{d}$ is the SLD for the diagonal density operator $\rho_{d}$ that is implicitly defined via the Lyapunov Equation (21) in Section 3.2. It is worth observing that $\tilde{L}\neq L_{\mathrm{coh}}$, since the Lyapunov equation represents a nonlinear transformation for a density operator ρ. Such a feature becomes evident by expanding $2\left(\partial_{\beta }\left(\rho_{d}\right)+\partial_{\beta }\left(\rho_{\mathrm{coh}}\right)\right)=2\partial_{\beta }\left(\rho \right)=\left\{\rho ,L\right\}=\left\{\left(\rho_{d}+\rho_{\mathrm{coh}}\right),\left(L_{d}+\tilde{L}\right)\right\}$, which leads to the relation

(35) $$\left\{\rho_{\mathrm{coh}},L_{\mathrm{coh}}\right\}=\left\{\rho_{\mathrm{coh}},\tilde{L}\right\}+\left(\left\{\rho_{\mathrm{coh}},L_{d}\right\}+\left\{\rho_{d},\tilde{L}\right\}\right),$$

which is evidently different from $\left\{\rho_{\mathrm{coh}},\tilde{L}\right\}$. The derivative $\partial_{\beta }\left(\rho_{\mathrm{coh}}\right)$ is a hollow operator by definition, and $\left\{\rho_{\mathrm{coh}},L_{d}\right\}$ is hollow due to Proposition 1. Hence, $\left\{\rho ,\tilde{L}\right\}=\left\{\rho_{\mathrm{coh}},\tilde{L}\right\}+\left\{\rho_{d},\tilde{L}\right\}$ is also a hollow operator.

We focused on the operator $\tilde{L}$ given its importance for the computation of the QFI $F\left(\rho ,\beta \right)=\mathrm{Tr}\left[\partial_{\beta }\left(\rho \right)L\right]=\mathrm{Tr}\left[\rho L^{2}\right]$. In fact, as provided in the proof at the end of this section, we find that the QFI F(ρ,β) can be decomposed as the corresponding “classical” Fisher information yielded by the diagonal density operator $\rho_{d}$, plus the extra non-negative term $\mathrm{tr}\left(\rho {\tilde{L}}^{2}\right)$:

(36) $$F\left(\rho ,\beta \right)=\mathrm{Tr}\left[\partial_{\beta }\left(\rho \right)L\right]=F\left(\rho_{d},\beta \right)+\mathrm{Tr}\left[\rho {\tilde{L}}^{2}\right],$$

where $F\left(\rho_{d},\beta \right)=\mathrm{Tr}\left[\partial_{\beta }\left(\rho_{d}\right)L_{d}\right]=\mathrm{Tr}\left[\rho_{d}L_{d}^{2}\right]$ [see Equation (30)]. Once again, it is worth pointing out that $\mathrm{Tr}[\rho {\tilde{L}}^{2}]\geq 0$ for any time *t* due to the positive semidefiniteness of ρ and ${\tilde{L}}^{2}$; indeed, the eigenvalues of ρ and ${\tilde{L}}^{2}$ are non-negative. Therefore, in conclusion, the QFI acquired by a generic quantum state ρ about the inverse temperature β of a thermal bath is always *greater than or equal* to the information acquired by a diagonal density operator $\rho_{d}$ whose diagonal elements are the same as those of ρ. Interestingly, this analysis is not specific to the thermalization dynamics in Section 2 but holds for a generic open quantum map. In the next section, we show the analytical expression of $\tilde{L}$ for qubit thermometers [29,35,36,37,38].

**Proof of** **Equation (36).** In order to determine the expression of the QFI $F\left(\rho ,\beta \right)=\mathrm{Tr}\left[\partial_{\beta }\left(\rho \right)L\right]$, let us evaluate the terms composing $\partial_{\beta }\left(\rho \right)L$, recalling that $\rho =\rho_{d}+\rho_{\mathrm{coh}}$ and $L=L_{d}+\tilde{L}$:

$$\begin{array}{ccc} \;2\partial_{\beta }\left(\rho \right)L & = & \left\{\rho ,L\right\}L=\{\left(\rho_{d}+\rho_{\mathrm{coh}}\right),L_{d}+\tilde{L}\}L\\ & = & \left(\left\{\left(\rho_{d}+\rho_{\mathrm{coh}}\right),L_{d}\right\}+\left\{\left(\rho_{d}+\rho_{\mathrm{coh}}\right),\tilde{L}\right\}\right)L\\ & = & \left\{\rho_{d},L_{d}\right\}L+\left\{\rho_{\mathrm{coh}},L_{d}\right\}L+\left\{\rho_{d},\tilde{L}\right\}L+\left\{\rho_{\mathrm{coh}},\tilde{L}\right\}L\\ & = & \left\{\rho_{d},L_{d}\right\}L+\left\{\rho_{\mathrm{coh}},L_{d}\right\}L+\left\{\rho ,\tilde{L}\right\}L\\ & = & \left\{\rho_{d},L_{d}\right\}L_{d}+\underset{*}{\underset{︸}{\left\{\rho_{d},L_{d}\right\}\tilde{L}}}+\underset{\mathrm{hollow}\;\mathrm{via}\;1}{\underset{︸}{\left\{\rho_{\mathrm{coh}},L_{d}\right\}L_{d}}}+\underset{*}{\underset{︸}{\left\{\rho_{\mathrm{coh}},L_{d}\right\}\tilde{L}}}+\underset{\mathrm{hollow}\;\mathrm{via}\;1}{\underset{︸}{\left\{\rho ,\tilde{L}\right\}L_{d}}}+\left\{\rho ,\tilde{L}\right\}\tilde{L}\;. \end{array}$$

As also pointed out in the formula, the terms $\left\{\rho_{\mathrm{coh}},L_{d}\right\}L_{d}$ and $\left\{\rho ,\tilde{L}\right\}L_{d}$ are hollow matrices according to Proposition 1. Thus, their trace is identically equal to zero. Moreover, by summing the terms identified with ∗, one obtains $\left\{\rho_{d},L_{d}\right\}\tilde{L}+\left\{\rho_{\mathrm{coh}},L_{d}\right\}\tilde{L}=\left\{\rho ,L_{d}\right\}\tilde{L}$, whereby $\mathrm{Tr}\left[\left\{\rho ,L_{d}\right\}\tilde{L}\right]=\mathrm{Tr}\left[\left\{\rho ,\tilde{L}\right\}L_{d}\right]=0$ due to using the cyclic property of the trace and again Proposition 1. As a result,

$$\begin{array}{ccc} F(\rho ,\beta ) & = & \mathrm{Tr}\left[\partial_{\beta }\left(\rho \right)L\right]=\frac{1}{2}\mathrm{Tr}\left[\left\{\rho_{d},L_{d}\right\}L_{d}\right]+\frac{1}{2}\mathrm{Tr}\left[\left\{\rho ,\tilde{L}\right\}\tilde{L}\right]=\\ & = & \mathrm{Tr}\left[\rho_{d}L_{d}^{2}\right]+\mathrm{Tr}\left[\rho {\tilde{L}}^{2}\right]\;. \end{array}$$

□

## 4. Metrological Limits of Qubit Thermometers: Analytical Derivation

In this section, we specialize the treatment of the Quantum Fisher Information for an *N*-level quantum system, developed in Section 3, to the case of a qubit thermometer (N=2). In Section 4.1, we derive the full analytic expression of the QFI for a qubit state. Afterward, we discuss in detail the effects on finite-time quantum thermometry from initializing the thermometer in a diagonal state (Section 4.2) and then the role played by quantum coherence (Section 4.3).

### 4.1. Derivation of the QFI

Let us consider a generic density operator ρ(0) for a qubit, parameterized by (a,r,ϕ)∈[0,1]×[0,1]×[0,2π]:

(37) $$\rho \left(0\right)=\left[\begin{array}{ccc} 1-a & \; & \sqrt{(1-a)a}\;r\;e^{i\phi }\\ \sqrt{(1-a)a}\;r\;e^{-i\phi } & \; & a \end{array}\right].$$

Thus, a qubit thermometer with initial state ρ(0), undergoing the thermalization dynamics from Equation (1) with population terms as in Equation (14) and with coherence decay rate $c_{12}=c_{21}=\frac{1}{2}\left(\Gamma_{12}+\Gamma_{21}\right)=-\frac{1}{2}\lambda =-\frac{\gamma }{2}{\left(\pi_{2}-\pi_{1}\right)}^{-1}$, evolves as

(38) $$\begin{array}{cc} \rho_{\beta }\left(t\right) & =\left[\begin{array}{ccc} 1-\pi_{2}+e^{\lambda t}\left(\pi_{2}-a\right) & \; & e^{\frac{1}{2}\lambda t}e^{i\omega_{12}t}\rho_{12}\left(0\right)\\ e^{\frac{1}{2}\lambda t}e^{-i\omega_{12}t}\rho_{12}^{*}\left(0\right) & \; & \pi_{2}-e^{\lambda t}\left(\pi_{2}-a\right) \end{array}\right]=\\ & \equiv \left[\begin{array}{ccc} 1-\rho_{22}\left(t,\beta \right) & \; & \rho_{12}\left(t,\beta \right)\\ \rho_{12}^{*}\left(t,\beta \right) & \; & \rho_{22}\left(t,\beta \right) \end{array}\right]. \end{array}$$

To study the QFI of the thermometer’s state $\rho_{\beta }\left(t\right)$ about β, we need to compute the Symmetric Logarithmic Derivative $L_{\beta }\left(t\right)$ of $\rho_{\beta }\left(t\right)$, as in Equation (21). To do so, we compute $\partial_{\beta }\left(\rho_{\beta }\left(t\right)\right)$ using Equations (16) and (17):

(39) $$\begin{array}{cc} \partial_{\beta }\left(\rho_{\beta }\left(t\right)\right) & =\left[\begin{array}{ccc} -\left(1-\pi_{2}\right)\pi_{2}\;\omega_{12}\;\delta \left(t,\beta ,a\right) & \; & \alpha \rho_{12}\left(t,\beta \right)\\ \alpha \rho_{12}^{*}\left(t,\beta \right) & \; & \left(1-\pi_{2}\right)\pi_{2}\;\omega_{12}\;\delta \left(t,\beta ,a\right) \end{array}\right]=\\ & \equiv \left[\begin{array}{ccc} -\partial_{\beta }\left(\rho_{22}\left(t,\beta \right)\right) & \; & \alpha \rho_{12}\left(t,\beta \right)\\ \alpha \rho_{12}^{*}\left(t,\beta \right) & \; & \partial_{\beta }\left(\rho_{22}\left(t,\beta \right)\right) \end{array}\right], \end{array}$$

where δ(t,β,a) is defined by Equation (19). Thus, expanding Equation (21) and writing $L_{\beta }\left(t\right)\equiv \left[\begin{array}{ccc} \ell_{11}\left(t,\beta \right) & & \ell_{12}\left(t,\beta \right)\\ \ell_{12}^{*}\left(t,\beta \right) & & \ell_{22}\left(t,\beta \right) \end{array}\right]$, we end up with the following three equations for three unknowns:

(40) −∂βρ22(t,β)=1−ρ22(t,β)ℓ11(t,β)+Reρ12*(t,β)ℓ12(t,β)

(41) ∂βρ22(t,β)=ρ22(t,β)ℓ22(t,β)+Reρ12*(t,β)ℓ12(t,β)

(42) $$\begin{array}{cc} 2\alpha \;\rho_{12}\left(t,\beta \right) & =\left(\ell_{11}\left(t,\beta \right)+\ell_{22}\left(t,\beta \right)\right)\rho_{12}\left(t,\beta \right)+\ell_{12}\left(t,\beta \right). \end{array}$$

Solving Equations (40)–(42) as a function of $\ell_{11}\left(t,\beta \right)$, $\ell_{22}\left(t,\beta \right)$ and $\ell_{12}\left(t,\beta \right)$ leads us to:

(43) $$\begin{array}{cc} \ell_{11} & =\frac{2\partial_{\beta }\left(\rho_{22}\right)|\rho_{12}{|}^{2}-2\alpha \;\rho_{22}{\left|\rho_{12}\right|}^{2}-\rho_{22}\;\partial_{\beta }\left(\rho_{22}\right)}{\left(1-\rho_{22}\right)\rho_{22}-{\left|\rho_{12}\right|}^{2}} \end{array}$$

(44) $$\begin{array}{cc} \ell_{22} & =\frac{-2\partial_{\beta }\left(\rho_{22}\right)|\rho_{12}{|}^{2}-2\alpha \left(1-\rho_{22}\right){\left|\rho_{12}\right|}^{2}+\left(1-\rho_{22}\right)\partial_{\beta }\left(\rho_{22}\right)}{\left(1-\rho_{22}\right)\rho_{22}-{\left|\rho_{12}\right|}^{2}} \end{array}$$

(45) $$\begin{array}{cc} \ell_{12} & =\frac{2\alpha \left(1-\rho_{22}\right)\rho_{22}-\left(1-2\rho_{22}\right)\partial_{\beta }\left(\rho_{22}\right)}{\left(1-\rho_{22}\right)\rho_{22}-{\left|\rho_{12}\right|}^{2}}\;\rho_{12}\;. \end{array}$$

As a result, the QFI at time *t* of the thermometer’s state $\rho_{\beta }\left(t\right)$ about β is

(46) F(ρβ(t),β)=Tr∂βρβ(t)Lβ(t)=∂βρ22ℓ22−ℓ11+2αReρ12*ℓ12==4α|ρ12|2α(1−ρ22)ρ22+(1−2ρ22)∂βρ22+∂βρ2221−4|ρ12|2(1−ρ22)ρ22−|ρ12|2==∂βρ222(1−ρ22)ρ22−|ρ12|2+4|ρ12|2α2(1−ρ22)ρ22+α(1−2ρ22)∂βρ22−∂βρ222(1−ρ22)ρ22−|ρ12|2,

where α is given by Equation (18), and all the elements of $L_{\beta }\left(t\right)$ and $\rho_{\beta }\left(t\right)$ in Equations (43)–(46) depend on *t* and β (albeit not explicitly written).

Now, some remarks are in order. (1) If the initial density operator ρ(0) is diagonal (i.e., r=0), then $\rho_{12}\left(t,\beta \right)=0$ for any time *t* and the QFI of $\rho_{\beta }\left(t\right)$ about β is equal to the QFI of the diagonal density operators with elements p(t,β,a) obtained by initializing the quantum thermometer in diag(1−a,a). (2) The QFI $F(\rho_{\beta }\left(t\right),\beta )=0$ at t=0, since α(0,β)=0 and δ(0,β,a)=0 for any a,β. Thus, as expected, measuring the state of the quantum thermometer at t=0 yields no information on the inverse temperature of the thermal bath, as the initial state of the thermometer is β-independent. (3) For t→∞, δ→1 and $|\rho_{12}|$ vanishes exponentially fast; hence, the QFI is converging to a β-dependent value that is the thermal variance of the Hamiltonian *H* [see Equation (29)]. (4) The diagonal and off-diagonal elements of the quantum thermometer’s state always refer to the Hamiltonian *H*. Thus, the latter implicitly represents the observable of choice for projective measurements (i.e., projections on the eigenbasis $\{|\epsilon_{1}\rangle \;\langle \epsilon_{1}\left|,\right|\epsilon_{2}\rangle \;\langle \epsilon_{2}|\}$) to carry out thermometry in a nonequilibrium regime. It is the optimal solution, over all the possible measurement observables (even β-dependent) if the initial state of the thermometer is diagonal in *H*.

At this point, it is worth making an additional remark on the role of the QFI in providing a bound for quantifying the precision Δβ while estimating β, in comparison with the concept of *accuracy*. The precision, as mentioned previously, follows from the Cramér–Rao bound [see Equation (23)], while the accuracy is defined upon defining an estimator for the parameter β. A possible choice for the latter in qubit thermometers can be given by $\beta_{\mathrm{eff}}\left(t\right)\equiv -\frac{1}{\omega_{12}}\log\left(\rho_{22}\left(t\right)/\rho_{11}\left(t\right)\right)$ that returns the effective inverse temperature associated with the density operator of the thermometer at any time *t*. To compute $\beta_{\mathrm{eff}}\left(t\right)$, full access to $\rho_{\beta }\left(t\right)$ is needed. However, for any β-estimator β(t) one wishes to consider, the estimation accuracy is defined by the quantity

(47) $$\delta \beta \left(t\right)\equiv \left|\beta (t)-\beta \right|,$$

meaning that the lower δβ(t) becomes, the higher the accuracy. If we consider again $\beta_{\mathrm{eff}}\left(t\right)$ (just to clarify the difference between precision and accuracy), then it converges asymptotically to the inverse temperature β: $\beta_{\mathrm{eff}}\left(t\right)\to \beta$ as t→∞. Therefore, the estimation error δβ(t) tends to 0, implying that asymptotically $\beta_{\mathrm{eff}}\left(t\right)$ is indeed an unbiased estimator of β. Conversely, the precision depends on the QFI that, as expressed by Equation (27), tends asymptotically to Var${\left(H\right)}_{\rho_{\beta }}$. This poses a finite limit to the precision of the estimation: Δβ>0. Since $\beta_{\mathrm{eff}}\left(t\right)$ tends to 0 asymptotically, we can see that the estimation of β falls well within the accuracy limits given by Δβ. We observed that this is indeed the case also for all finite times *t*, namely δβ(t)<Δβ(t).

### 4.2. The Role of the Diagonal Elements in the Initial Density Operator

To analyze the time behavior of the QFI, we start by considering initial density operators with only diagonal elements *a* and 1−a (thus r=0), with a∈[0,1]. In this regard, it is worth noting that one can determine three different regions for the QFI depending on the value of the temperature of the thermal bath, and the parameter *a* that characterizes the initial state of the qubit thermometer.

**Figure 1:**
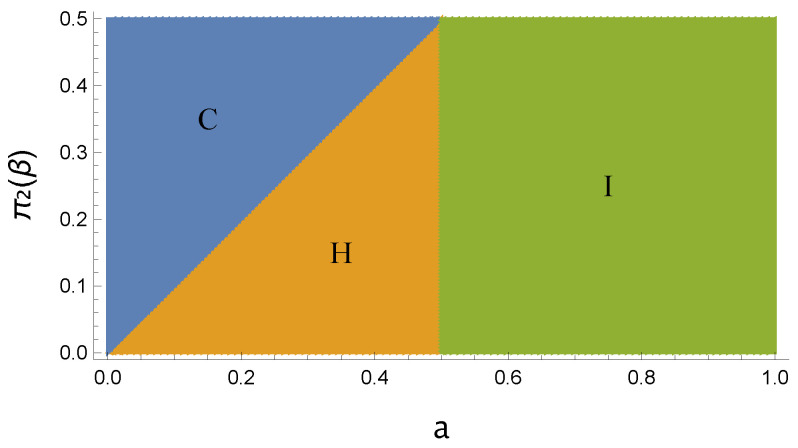
Pictorial representation of the parameter region identifying three distinct time behaviors of the QFI depending on the excited state population a∈[0,1] of the thermometer initial state (x-axis), and on the bath’s excited state population $\pi_{2}\left(\beta \right)\in \left[0,\frac{1}{2}\right]$ (y-axis). The three distinct regions are labeled C, H, and I referring to the thermometer being initialized, respectively, at a temperature colder than the bath, hotter than the bath, or inverted.

Once fixed, the energies $\epsilon_{1}$ and $\epsilon_{2}$ of the thermometer, the temperature β (taken as positive) uniquely defines the thermal probability $\pi_{2}\left(\beta \right)$, as well as $\pi_{1}\left(\beta \right)=1-\pi_{2}\left(\beta \right)$. For the scope of our analysis, $\pi_{2}\left(\beta \right)$ is considered to belong to the interval $\left[0,\frac{1}{2}\right]$. Moreover, for a qubit, any density operator with diagonal elements can be written as a thermal state. Accordingly, if $a\in [0,\pi_{2}]$, then the initial state of the thermometer is associated with a thermal distribution with a colder temperature than π. This fact gives a specific behavior to the time evolution of the QFI. In Figure 1, the region of the parameter space corresponding to $a\in [0,\pi_{2}]$ is denoted as ‘Region C’.

Then, any initial density operator with $a\in [\pi_{2},\frac{1}{2}]$ can be regarded as a thermal state with a hotter temperature than π; in Figure 1, we denote such a parameter region as ‘Region H’.

Finally, any initial diagonal state of the qubit thermometer with $a\in [\frac{1}{2},1]$ can be related to a thermal state with an ‘effective negative temperature’ that simply stands for a population-inverted state, so that the excited state is more populated than the ground state. This region is denoted as ‘Region I’ in Figure 1.

**Figure 2:**
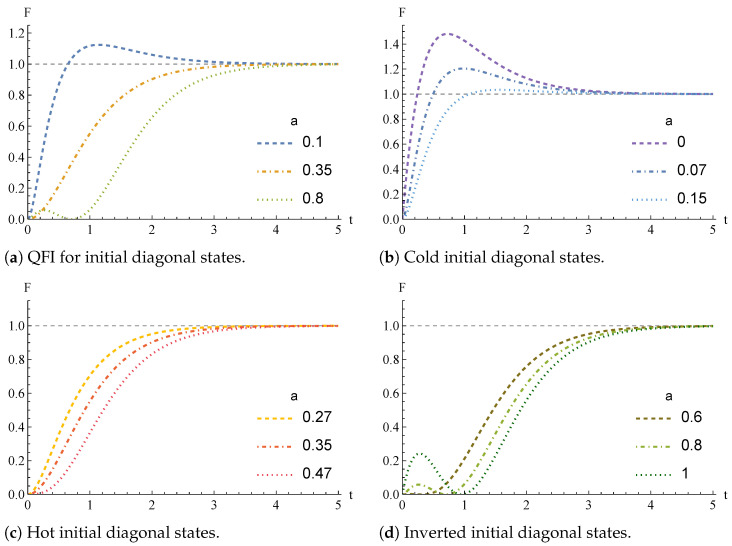
Time behavior of the QFI for initial diagonal density operators, showing the different regimes represented by the regions C, H, and I in Figure 1. In all figures $\pi_{2}=0.25$. Time *t* is measured in units of $\gamma^{-1}$. In each panel, *F* is normalized to its asymptotic value. (**a**) Time behavior of the QFI for initial diagonal density operators with a∈{0.1,0.35,0.8}. Each of the values of *a* is related to a different region: respectively, the regions C, H, and I are depicted in Figure 1. (**b**) Time behavior of the QFI for initial diagonal states colder than the thermal state π (region C in Figure 1). (**c**) Time behavior of the QFI initial diagonal states hotter than the thermal state π, (region H in Figure 1). (**d**) Time behavior of the QFI for inverted initial diagonal states (region I in Figure 1).

Let us now show the distinct behaviors for the time evolution of the QFI *F* in the three regions, which we plot altogether in Figure 2 as a function of *a* taking $\omega_{12}=1$, γ=1, and $\pi_{2}\left(\beta \right)=0.25$ (all these quantities are expressed in dimensionless units). We detail the curves referring to ‘Region C’ in Figure 2a, where we can observe that the QFI increases monotonically until it reaches a global maximum at some finite time $t^{*}$. After such a time, *F* decreases monotonically to the asymptotic value corresponding to the thermal fixed point of the thermalization dynamics. In all the panels of Figure 2, the QFI is normalized to such an asymptotic value, which is always the same independently of the initial state of the thermometer. In Figure 2b, the colder the initial state (i.e., the smaller the value of *a* with respect to $\pi_{2}\left(\beta \right)$), the greater the maximum value of the Fisher information that occurs at the early time $t^{*}$.

On the other hand, by initializing the qubit thermometer using parameters lying in the ‘Region H’, *F* increases monotonically from 0 to the asymptotic value, as shown in Figure 2c. The hotter the initial state (i.e., the greater the value of *a* in the interval $\left[\pi_{2},\frac{1}{2}\right]$), the slower the convergence of *F* to the asymptotic value.

We also detail in Figure 2d the time evolution of the QFI considering an initial inverted state for the qubit thermometer. In such a case, *F* increases until a local maximum, then decreases to zero, after which it monotonically increases again until the asymptotic value.

### 4.3. The Role of Coherence in the Initial Density Operator

Having analyzed the evolution of the QFI *F* in time for different initial diagonal states, we now study the role of quantum coherence in the initial state of a qubit thermometer. In doing this, it is worth noting that the quantum coherence in the initial state affects the value of the QFI $F(\rho_{\beta }\left(t\right),\beta )$, Equation (46), via the term $|\rho_{12}{\left(t,\beta \right)|}^{2}$. Thus, the phase ϕ entering the coherence term $\sqrt{(1-a)a}\;r\;e^{i\phi }$ in ρ(0) does not influence the QFI. Hence, we initialize the thermometer in a pure quantum state of the form given by Equation (37) with r=1 and ϕ=0. Note that for N>2, there will be multiple coherence terms, thus multiple relative phases, and the fact that the QFI does not depend on them may not be true in general.

**Figure 3:**
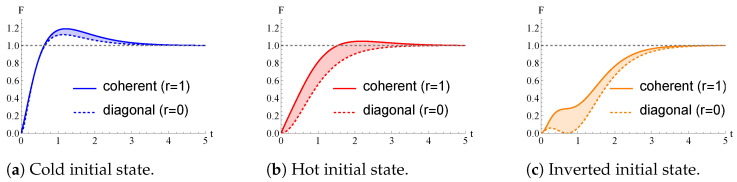
QFI $F(\rho_{\beta }\left(t\right),\beta )$, as a function of time *t*, with $\pi_{2}\left(\beta \right)=0.25$. We compare the different behaviors given from initializing the qubit thermometer in a diagonal initial density operator, r=0, or in a pure initial state, r=1, which bears some quantum coherence. From panel to panel, we vary the diagonal elements in the initialization of the thermometer. Time *t* is measured in units of $\gamma^{-1}$. In each panel, *F* is normalized to its asymptotic value. (**a**) Cold initial state; diagonal elements: a=0.1.; (**b**) Hot initial state; diagonal elements: a=0.35.; (**c**) Inverted initial state; diagonal elements: a=0.8.

In Figure 3, we plot the time behavior of $F(\rho_{\beta }\left(t\right),\beta )$ for pairs of initial quantum states given by a pure state (r=1) and a diagonal one (r=0) with the same diagonal elements (thus, the same value of *a*). As a result, for the same value of *a*, setting r=1 (meaning that quantum coherence is present in the initial state of the qubit thermometer) instead of r=0 brings an advantage in terms of the QFI maximization at finite times. Such an advantage due to quantum coherence decreases when *a* is small and vanishes if a=0. However, even with r=1, the best performances in terms of the magnitude of QFI for any time *t* are obtained setting a=0, which refers to the ground state of $H_{0}$.

We conclude this section by stressing that, in the case the qubit thermometer is initialized in a pure state (r=1), the three distinct behaviors of the QFI over time outlined in Figure 1, namely $a\in [0,\pi_{2}]$ (Region C), $a\in [\pi_{2},\frac{1}{2}]$ (Region H), and $a\in [\frac{1}{2},1]$ (Region I), are no longer valid in general, given that the QFI in Region H can be larger than 1.

## 5. Discussion

In this paper, we computed the QFI associated with a quantum thermometer in weak contact with a thermal bath. Maximizing the QFI allowed us to determine the optimal time, within the transient thermalization dynamics, and the initial state of the quantum thermometer such that the thermometry precision is enhanced. We specialized our analysis to the case of qubit thermometers, whereby analytical expressions are derived.

Now, we discuss the application of our results to an experimental platform. We consider the quantum optics setup in [38] where the dynamics of a qubit thermometer in interaction with a thermal bath is simulated. The thermometer Hamiltonian is $H=\hbar \omega \sigma_{z}/2$, so that $\epsilon_{2}=\hbar \omega /2$, $\epsilon_{1}=-\hbar \omega /2$, and thus, $\omega_{12}=\epsilon_{2}-\epsilon_{1}=\hbar \omega$.

The thermalization dynamics of the qubit in the experiments are described by a generalized amplitude damping channel, defined by the Kraus operators [38,39].

(48) $$\begin{array}{cc} K_{0} & =\sqrt{p_{1}}\left[\begin{array}{cc} 1 & 0\\ 0 & \sqrt{1-p_{2}} \end{array}\right],\;K_{1}=\sqrt{p_{1}}\left[\begin{array}{cc} 0 & \sqrt{p_{2}}\\ 0 & 0 \end{array}\right], \end{array}$$

(49) $$\begin{array}{cc} K_{2} & =\sqrt{1-p_{1}}\left[\begin{array}{cc} \sqrt{1-p_{2}} & 0\\ 0 & 1 \end{array}\right],\;K_{3}=\sqrt{1-p_{1}}\left[\begin{array}{cc} 0 & 0\\ \sqrt{p_{2}} & 0 \end{array}\right], \end{array}$$

where the probabilities $p_{1}$ and $p_{2}$ are $p_{1}=\frac{n_{12}}{2n_{12}-1}$ and $p_{2}=1-\exp\left(-\left(1+n_{12}\right)\tilde{\tau }\right)$, with $n_{12}$ being the usual thermal ratio, as in Equation (4). The quantity $\tilde{\tau }$ is the dimensionless time that is representative of the duration of the thermalization dynamics; in [38], $\tilde{\tau }$ is taken in the interval [0,0.3]. It is worth noting that $p_{1}$ is approximately equal to 1/2 ($p_{1}≃1/2$) for $n_{12}>5$ and that $p_{2}\approx \left(1+n_{12}\right)\tilde{\tau }$ for $n_{12}\tilde{\tau }<1$. Thus, comparing the Kraus operator $K_{1}$ and the jump operator $L_{12}$ in Section 2, in the first approximation, we can set $\Gamma_{12}\approx p_{1}p_{2}\omega_{12}$, where multiplying by $\omega_{12}$ allows to give $\Gamma_{12}$ the correct dimensionality of [time]^−1^ with ℏ=1. Accordingly, $\Gamma_{12}\approx \frac{\tilde{\tau }\omega_{12}}{2}\left(1+n_{12}\right)$, i.e., $\gamma \approx \frac{\tilde{\tau }\omega_{12}}{2}$. Using the generalized amplitude damping channel gives comparable results with respect to the ones provided by the model we introduced in Section 2.

The qubit thermometer is initialized in the pure state ρ(0)=|ψ〉 〈ψ| with |ψ〉=cos(θ/2)|0〉+sin(θ/2)|1〉, where |0〉 and |1〉 are the eigenstates of the Pauli matrix $\sigma_{z}$, and θ∈[0,2π]. The angle θ sets the magnitude of the quantum coherence in ρ(0), which is equal to cos(θ/2)sin(θ/2)=sin(θ)/2. In [38], |0〉 and |1〉 are the horizontal |H〉 and vertical |V〉 polarization states of the photons employed as thermometers, while θ is given by the birefringent angle of the spatial light modulator composing a Sagnac interferometer. According to the parametrization in (37), the initial state of the qubit thermometer realized in [38] is obtained by setting $a=\sin^{2}\left(\theta /2\right)$ [i.e., $\theta =2\arcsin(\sqrt{a})$], r=1 and ϕ=0.

After the initialization, the thermometer is put in contact with the thermal bath for a time τ, which varied in different experiments. This means that for each experiment, one chooses the time τ, then lets the thermometer interact with the thermal bath for the duration τ, and finally measures the state of the thermometer (via quantum state tomography), with the goal of determining the temperature of the bath.

In [38], a thermometry task is carried out by discriminating between two different values of $n_{12}=1/\left(e^{\beta \omega_{12}}-1\right)$ (dimensionless number): $n_{12}^{\left(c\right)}=5.5$ and $n_{12}^{\left(h\right)}=9.5$, corresponding, respectively, to the effective temperatures of a cold and hot thermal bath. Hence, the inverse temperature β as a function of $n_{12}$ is $\beta =\frac{1}{\omega_{12}}\ln\left(\frac{1+n_{12}}{n_{12}}\right)$, so that $\beta_{h}\approx 0.020$ and $\beta_{c}\approx 0.033$ by choosing $\omega_{12}=5$.

Referring to the experimental setting in [38], we can determine both the optimal value of θ in the initial state of the thermometer and the optimal time $t^{*}$ at which performing the thermometry, such that the analytical expression of the QFI we computed in Section 4 is maximized. This is useful since the maximization of the QFI leads to enhancing the precision in estimating the value of β. Thus, let us set $\omega_{12}=5$ (i.e., $\epsilon_{1}=-\omega_{12}/2=-2.5$, $\epsilon_{2}=\omega_{12}/2=2.5$, $\beta_{h}\approx 0.020$, and $\beta_{c}\approx 0.033$) and $\tilde{\tau }=0.05$ so that γ≈0.125. With this choice of parameter values, we have that $\lambda_{j}=\gamma {\left(\pi_{2}\left(\beta_{j}\right)-\pi_{1}\left(\beta_{j}\right)\right)}^{-1}$, with $\pi_{k}\left(\beta_{j}\right)=e^{-\beta_{j}\epsilon_{k}}/Z_{\beta_{j}}$, entering in the analytical expressions of $\alpha (t,\beta_{j})$ and $\delta (t,\beta_{j},a\left(\theta \right))$ of Equations (18) and (19) with j=c,h.

**Figure 4:**
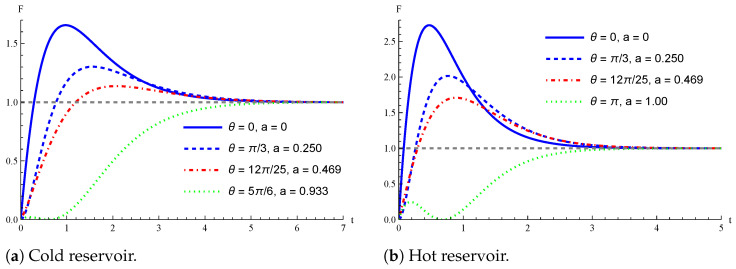
QFI over time by initializing the qubit thermometer in the density operator (37) with r=1, ϕ=0, and $a=\sin^{2}\left(\theta /2\right)$. We also set $\omega_{12}=5$ and $\tilde{\tau }=0.05$. Moreover, in panel (**a**), cold reservoir, $\beta_{c}=0.0334$, we consider θ=0,π/3,12π/25,5π/6, and $n_{12}=5.5$, which means $\beta_{c}=0.0334$. Conversely, in panel (**b**) hot reservoir, $\beta_{h}\approx 0.020$, we take θ=0,π/3,12π/25,π, and $n_{12}=9.5$, leading to $\beta_{h}\approx 0.020$.

In Figure 4, we plot the QFI $F(\rho_{\beta }\left(t\right),\beta )$ of Equation (46) as a function of time, for θ=0,π/3,12π/25,5π/6 [panel (a)] and θ=0,π/3,12π/25,π [panel (b)]. The two panels of Figure 4 differ for the value of the inverse temperature: $\beta_{c}\approx 0.033$ in panel (a) and $\beta_{h}\approx 0.020$ in panel (b). In both cases, the greatest value of the QFI is obtained by setting θ=0 (i.e., a=0) in the initial transient dynamics. For the experiments in [38], θ=0 corresponds to initializing the single photons encoding the qubit thermometer in the horizontal or vertical polarization state. Interestingly, there are time intervals (before the qubit is fully thermalized) where the QFI is not maximized by initializing the qubit thermometer in the ground state of $H_{0}$, i.e., by setting θ=0.

**Figure 5:**
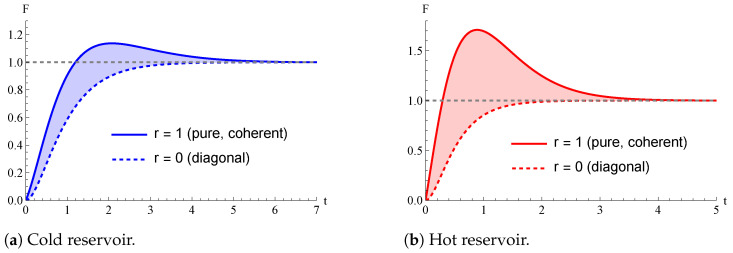
Role of coherence in the initial state. QFI over time in the realistic scenario realized in [38]. The qubit thermometer is initialized in the density operator (37) with r=1 or r=0. As in Figure 4, we have in panel (**a**) cold reservoir, $\beta_{c}=0.0334$, $n_{12}=5.5$, and $\beta_{c}=0.0334$, while in panel (**b**), hot reservoir, $\beta_{h}\approx 0.020$, $n_{12}=9.5$, and $\beta_{h}\approx 0.020$. In both panels, we set ϕ=0, θ=12π/25, thus a=0.469, $\omega_{12}=5$ and $\tilde{\tau }=0.05$.

We conclude by showing in Figure 5 the advantage entailed by the presence of quantum coherence in the initial state of the thermometer. In agreement with Figure 3, we can observe an apparent advantage for both the cold and hot temperatures considered in [38]. Moreover, it is also evident that, as long as r=0, the three time behaviors of the QFI in the regions C, H, and I described in Figure 1 are recovered. This is not necessarily true by initializing the qubit thermometer in a pure state (r=1).

For the sake of interpretation, the presence of quantum coherence in the thermometer’s state entails a higher purity of the state itself. The latter can be mapped unitarily to a diagonal density operator whose elements can be linked to a smaller effective temperature. Thus, from this point of view, the increase in the QFI could be seen as a cooling effect on the thermometer’s state due to quantum coherence, provided the amount of purity is kept the same (a unitary mapping is indeed assumed).

Based on these results, further experimental tests are foreseeable, provided the availability of an estimation method/algorithm that returns the estimated inverse temperature using the density operator of the qubit thermometer (to be obtained via tomography) in the time interval where the QFI is maximized. In this way, one could explore if the presence of quantum coherence in the initial states of the thermometer shows up even in a smaller estimation error in a given nonequilibrium regime.

Furthermore, it is worth studying the metrological limits of quantum thermometry with a multiqubit thermometer, under the assumption that each qubit thermometer interacts weakly with the thermal bath. In this context, the question is still to determine how to initialize the multiqubit thermometer before it is put in contact with the bath. We expect that the key element to maximize the QFI, enhancing the precision for estimating the temperature of the bath, is the *correlation* of the global initial state of quantum thermometers. In fact, if the qubit thermometers are not correlated to each other, the value of the QFI increases linearly with the number of thermometers. This is because, in the weak coupling regime, each thermometer interacts independently with the bath, and the thermometers do not interact with each other through the bath itself. Conversely, we expect a superlinear scaling of the QFI in the number of quantum thermometers when their global quantum state is correlated. Interestingly, a similar result has been found in the setting of collision quantum thermometry [19,20,21].

Finally, we briefly consider the extension of our work to the case of a non-Markovian environment. From a formal point of view, our framework can readily be generalized to the case when the open system dynamics of the thermometer originated by a non-Markovian (e.g., time-dependent) master equation, provided that the weak thermometer-environment coupling limit is still valid. In this case, the assumption of negligible backaction on the environment could also still be considered. While the thermometer dynamics would be different from the one explored here, the framework we developed for quantum thermometry with the aim to attain a bound on its precision would still apply. From a more physical perspective, additional care would be required, since the more the environment becomes non-Markovian, the more it deviates from the concept of an ideal thermal bath. Therefore, the notion of its temperature may itself become a nontrivial issue. The case of a multiqubit thermometer in interaction with a non-Markovian environment would present similarities to the Markovian case, but only as far as the weak coupling limit holds. Should this not be the case, we foresee that additional elements would have to be included in the model, such as thermometer–environment correlations and intraenvironment interactions. Both in the single- and the multiqubit thermometry case, beyond the weak-coupling limit, other approaches need to be employed, such as those explored again in collision quantum thermometry.

## Data Availability

The original contributions presented in the study are included in the article, further inquiries can be directed to the corresponding author/s.

## Appendix A. On the Eigenvalues of the Transition Matrix *A_β_*

In this Appendix, we show that the transition matrix $A_{\beta }$, from Equation (7), has a single null eigenvalue, whereas the remaining N−1 eigenvalues have a negative real part. Furthermore, we show that the eigenvector with null eigenvalue is the thermal population π(β).

In continuous-time Markov chain theory, the state of a Markov chain at time *t* is described by a probability vector p(t), where $p_{i}\left(t\right)$ is the probability of the chain being in state *i*. A Markov process is characterized by a transition rate matrix *A*, where the off-diagonal entries $a_{ij}$ (i≠j) denote the instantaneous transition rate from state *j* to state *i*. The evolution of the process is given by the differential equation $\dot{p}\left(t\right)=A\;p\left(t\right)$ or the equivalent integral equation $p\left(t\right)=e^{A(t-t_{0})}p\left(t_{0}\right)$.

For the thermalization model in Section 2, the transition matrix $A_{\beta }$, which describes the evolution of the population terms, exhibits the following properties:

1. $A_{\beta }={\left[a_{ij}\right]}_{ij}$ is a real square matrix, that is, $A_{\beta }\in R^{N\times N}$.
2. The off-diagonal elements are positive, that is, $a_{ij}>0$$\forall_{i\neq j}$.
3. The columns sum to zero, that is, $\sum_{i=1}^{N}a_{ij}=0$∀j, as a direct consequence of probability conservation, see Equations (5) and (9).

To prove that the transition matrix $A_{\beta }$ has only one null eigenvalue and remaining N−1 eigenvalues with a negative real part, we first show that all the eigenvalues have a nonpositive real part, and then demonstrate that there is only one null eigenvalue.

### Appendix A.1. The Real Part of the Eigenvalues of A_β_ Is Nonpositive

To study the eigenvalues of $A_{\beta }$, we introduce the following theorem:

**Theorem** **A1** (Gershgorin circle theorem [46])**.** *Let A be a complex N×N matrix. Define $R_{j}$ as the sum of the absolute values of the nondiagonal entries in the $j^{th}$ column: $R_{j}=\sum_{\begin{array}{c} i=1\\ i\neq j \end{array}}^{N}\left|a_{ij}\right|.$*
*Let $D\left(a_{jj},R_{j}\right)=\{z\in C:|z-a_{jj}\left|\leq R_{j}\right\}$ be the closed disc centered at $a_{jj}$ with radius $R_{j}$. Such a disk is called a Gershgorin disk. Then each eigenvalue of A lies within at least one of the Gershgorin discs $D(a_{jj},R_{j})$.*

Applying the Gershgorin circle theorem to our matrix $A_{\beta }$, together with Properties 2 and 3 above, each eigenvalue ν must satisfy, for at least one j∈{1,…,N}, the following inequality:

(A1) $$\begin{array}{c} |\nu \;-\;a_{jj}|\leq \sum_{\begin{array}{c} i=1\\ i\neq j \end{array}}^{N}|a_{ij}|=-a_{jj}=\left|a_{jj}\right|, \end{array}$$

which implies that any eigenvalue of $A_{\beta }$ has a nonpositive real part.

### Appendix A.2. A_β_ Has a Single Null Eigenvalue

The existence of a null eigenvalue of $A_{\beta }$ follows immediately from the fact that the columns of the matrix sum up to 0. To show this, construct an auxiliary N×N matrix *B*, equal to $A_{\beta }$, but with the last row zeroed. Since row operations do not change the nullity of a matrix, *B* has the same number of null eigenvalues as $A_{\beta }$.

(A2) $$\begin{array}{c} B=\left[\begin{array}{cccc} a_{11} & \cdots & a_{1(N-1)} & a_{1N}\\ \vdots & \ddots & \vdots & \vdots \\ a_{1(N-1)} & \cdots & a_{\left(N-1)(N-1\right)} & a_{\left(N-1)(N-1\right)}\\ 0 & \cdots & 0 & 0 \end{array}\right] \end{array}$$

Construct also a second auxiliary (N−1)×(N−1) matrix *C* by removing the last row and column of *B*. Note that each eigenvalue λ of *C* is also an eigenvalue of *B* since you can construct eigenvectors of *B* by padding the eigenvectors of *C* with a 0:

$$\begin{array}{cc} \; & \mathrm{Let}\;v=[v_{1}\;\cdots \;v_{N-1}]\;\;\mathrm{such}\;\mathrm{that}\;Cv=\lambda v;\\ \; & \mathrm{denote}\;w=[v_{1}\;\cdots \;v_{N-1}\;0]\;⟹\;Bw=\lambda w. \end{array}$$

Once again, use the Gershgorin circle theorem to find that each eigenvalue of *C* has a strictly negative real part:

(A3) $$\begin{array}{c} |\lambda -a_{jj}|\leq \sum_{\begin{array}{c} i=1\\ i\neq j \end{array}}^{N-1}|a_{ij}|=|a_{jj}|-a_{Nj}<\left|a_{jj}\right|. \end{array}$$

*C* has no null eigenvalues, thus *B* and, consequently, $A_{\beta }$ have only one null eigenvalue, which completes the proof.

### Appendix A.3. Proof of A_β_ **π** = 0

The verification of the thermal distribution π(β) as the null eigenvector of $A_{\beta }$ can be checked via direct substitution. For this purpose, we rewrite the entries $a_{ij}$ of $A_{\beta }$, Equations (8) and (9), as a function of the thermal population $\pi_{k}=e^{-\beta \epsilon_{k}}/Z_{\beta }$ with $Z_{\beta }=\sum_{k}e^{-\beta \epsilon_{k}}$:

(A4) $$\begin{array}{cc} n_{ij} & ={\left(e^{\beta \omega_{ij}}-1\right)}^{-1}={\left(\frac{\pi_{i}}{\pi_{j}}-1\right)}^{-1}=\frac{\pi_{j}}{\pi_{i}-\pi_{j}}, \end{array}$$

(A5) $$\begin{array}{cc} a_{ij}=\Gamma_{ij} & =\gamma \left\{\begin{array}{cc} n_{ij}+1 & =\pi_{i}/\left(\pi_{i}-\pi_{j}\right)\;\mathrm{for}\;i<j\\ n_{ji} & =\pi_{i}/\left(\pi_{j}-\pi_{i}\right)\;\mathrm{for}\;i>j\;. \end{array}\right. \end{array}$$

Notice that the off-diagonal elements of the transition matrix verify the detailed balance equation $a_{ij}\pi_{j}=a_{ji}\pi_{i}$ defined over the terms of the thermal distribution π. In fact,

(A6) $$\frac{a_{ij}}{a_{ji}}=\frac{\frac{\pi_{i}}{\pi_{i}-\pi_{j}}}{\frac{\pi_{j}}{\pi_{i}-\pi_{j}}}=\frac{\pi_{i}}{\pi_{j}}\;⟹\;a_{ij}\pi_{j}=a_{ji}\pi_{i}\;.$$

Finally, using the detailed balance Equation (A6) together with the fact that the columns of $A_{\beta }$ sum up to 0 (Property 3. at the beginning of this Appendix), we can prove that π is the eigenvector with null eigenvalue:

(A7) $$\begin{array}{c} a_{ij}\pi_{j}=a_{ji}\pi_{i}\Rightarrow A_{\beta }\pi ={\left[\begin{array}{c} \sum_{k}a_{ik}\pi_{k} \end{array}\right]}_{i}={\left[\begin{array}{c} \pi_{i}\sum_{k}a_{ki} \end{array}\right]}_{i}=0\;. \end{array}$$

## Appendix B. Derivatives of Thermometer’s State with Respect to the Inverse Temperature

### Appendix B.1. Derivation of Equations (16) and (17)

We aim to compute the derivatives of the population and coherence elements of a qubit thermometer, i.e.,

(A8) $$\begin{array}{cc} p(t,\beta ,a) & =\left(1-e^{\lambda t}\right)\pi +e^{\lambda t}p_{0}\left(a\right) \end{array}$$

(A9) $$\begin{array}{cc} \rho_{12}\left(t,\beta \right) & =e^{\lambda t/2}e^{i\;\omega_{12}t}\rho_{12}\left(0\right), \end{array}$$

with respect to β.

For this purpose, we start from calculating the derivative of the thermal population elements π:

(A10) $$\begin{array}{cc} \partial_{\beta }\left(\pi_{2}\right) & =\left(\epsilon_{1}\left(1-\pi_{2}\right)+\epsilon_{2}\pi_{2}-\epsilon_{2}\right)\pi_{2}=\left(1-\pi_{2}\right)\pi_{2}\;\omega_{12}\;, \end{array}$$

(A11) $$\begin{array}{cc} \partial_{\beta }\left(\pi_{1}\right) & =-\partial_{\beta }\left(\pi_{2}\right). \end{array}$$

Moreover, the derivative of the eigenvalue $\lambda ={\left(\pi_{1}-\pi_{2}\right)}^{-1}$ with respect to β is

(A12) $$\begin{array}{cc} \partial_{\beta }\left(\lambda \right) & =\partial_{\beta }\left({\left(\pi_{2}-\pi_{1}\right)}^{-1}\right)=2\lambda^{2}\partial_{\beta }\left(\pi_{1}\right)=-2\lambda^{2}\partial_{\beta }\left(\pi_{2}\right). \end{array}$$

Accordingly, the explicit expression for $\partial_{\beta }\left(p(t,\beta ,a)\right)$ reads as

(A13) $$\begin{array}{cc} \partial_{\beta }\left(p(t,\beta ,a)\right) & =\partial_{\beta }\left(\pi \right)-e^{\lambda t}\partial_{\beta }\left(\pi \right)-t\partial_{\beta }\left(\lambda \right)e^{\lambda t}\left(\pi -p_{0}\left(a\right)\right)=\\ \; & =\left[\begin{array}{c} \partial_{\beta }\left(\pi_{1}\right)\left(1-e^{\lambda t}-2t\lambda^{2}e^{\lambda t}\left(\pi_{1}-1+a\right)\right)\\ \partial_{\beta }\left(\pi_{2}\right)\left(1-e^{\lambda t}+2t\lambda^{2}e^{\lambda t}\left(\pi_{2}-a\right)\right) \end{array}\right]=\\ \; & =\left(1-\pi_{2}\right)\pi_{2}\;\omega_{12}\;\left(1-e^{\lambda t}+2t\lambda^{2}e^{\lambda t}\left(\pi_{2}-a\right)\right)\left[\begin{array}{c} -1\\ 1 \end{array}\right]=\\ \; & =\left(1-\pi_{2}\right)\pi_{2}\;\omega_{12}\;\delta \left(t,\beta ,a\right)\;\left[\begin{array}{c} -1\\ 1 \end{array}\right], \end{array}$$

where $\delta \left(t,\beta ,a\right)=1-e^{\lambda t}+2t\lambda^{2}e^{\lambda t}\left(\pi_{2}-a\right)$.

On the other hand, the explicit expression for $\partial_{\beta }\left(\rho_{12}\left(t,\beta \right)\right)$ is

(A14) $$\begin{array}{cc} \partial_{\beta }\left(\rho_{12}\left(t,\beta \right)\right) & =\frac{1}{2}t\;\partial_{\beta }\left(\lambda \right)e^{\lambda t/2}e^{i\;\omega_{12}t}\rho_{12}\left(0\right)=\\ \; & =\alpha \left(t,\beta \right)\;\rho_{12}\left(t,\beta \right), \end{array}$$

where $\alpha \left(t,\beta \right)=-\left(1-\pi_{2}\right)\pi_{2}\;\omega_{12}\lambda^{2}t$.

### Appendix B.2. Comment on the Difference between Partial and Total Derivatives

Let us consider a qubit thermometer, as well as the thermalization process that transforms a given input distribution $p_{0}\left(a\right)$ to an output distribution $p\left(t,\beta ,a\right):p_{0}\left(a\right)\to p\left(t,\beta ,a\right)$. Here, we take into account the dependence on β whenever necessary, being important for this discussion. In accordance with the thermalization dynamics in Section 2, the probability p(t,β,a) evolves as

(A15) $$p\left(t,\beta ,a\right)=\pi \left(\beta \right)-e^{\lambda (\beta )t}\left(\pi \left(\beta \right)-p_{0}\left(a\right)\right)=\left(1-e^{\lambda (\beta )t}\right)\pi \left(\beta \right)+e^{\lambda (\beta )t}p_{0}\left(a\right)\;,$$

where $\lambda \left(\beta \right)=\gamma {\left(\pi_{2}-\pi_{1}\right)}^{-1}<0$∀β guarantees the convergence of the system dynamics. In fact, as time increases, the distribution p(t,β,a) converges asymptotically to the thermal distribution $\pi \left(\beta \right)=\lim_{t\to \infty }p\left(t,\beta ,a\right)$. Such an asymptotic convergence occurs irrespectively of the initial distribution $p_{0}\left(a\right)$. Moreover, the derivative of p(t,β,a) with respect to β is given by

(A16) $$\begin{array}{cc} \partial_{\beta }\left(p(t,\beta ,a)\right) & =\partial_{\beta }\left(\pi \left(\beta \right)\right)-e^{\lambda (\beta )t}\partial_{\beta }\left(\pi \left(\beta \right)\right)-t\partial_{\beta }\left(\lambda \left(\beta \right)\right)e^{\lambda (\beta )t}\left(\pi \left(\beta \right)-p_{0}\left(a\right)\right)\;. \end{array}$$

We now analyze the case where, as input to the process, we set an initial distribution that is equal to the asymptotic distribution, i.e., $p_{0}\left(a\right)=\pi \left(\beta \right)$. The distributions $p_{0}\left(a\right)$ and π(β) are parameterized as

(A17) $$p_{0}\left(a\right)=\left[\begin{array}{c} 1-a\\ a \end{array}\right]\;\mathrm{and}\;\pi \left(\beta \right)=\left[\begin{array}{c} 1-\pi_{2}\left(\beta \right)\\ \pi_{2}\left(\beta \right) \end{array}\right].$$

If the initial distribution $p_{0}\left(a\right)$ is equal to the thermal distribution π(β), that is $a=\pi_{2}\left(\beta \right)$, then $p\left(t,\beta ,\pi_{2}\left(\beta \right)\right)$ is constant over time, as π(β) is a fixed point of the thermalization process. In particular,

(A18) $$p\left(t,\beta ,\pi_{2}\left(\beta \right)\right)=\pi \left(\beta \right)$$

and

(A19) $$\partial_{\beta }\left(p(t,\beta ,a)\right){|}_{a=\pi_{2}\left(\beta \right)}=\partial_{\beta }\left(\pi \left(\beta \right)\right)-e^{\lambda (\beta )t}\partial_{\beta }\left(\pi \left(\beta \right)\right)=\left(1-e^{\lambda (\beta )t}\right)\partial_{\beta }\left(\pi \left(\beta \right)\right)\;.$$

It is worth noting that the right-hand side of Equation (A19) is different from the partial derivative of the right-hand side of Equation (A18) with respect to β. Formally,

(A20) $$\partial_{\beta }\left(p(t,\beta ,a)\right){|}_{a=\pi_{2}\left(\beta \right)}\neq \partial_{\beta }\left(p\left(t,\beta ,\pi_{2}\left(\beta \right)\right)\right)=\partial_{\beta }\left(\pi \left(\beta \right)\right)\;.$$

The discrepancy in Equation (A20) can be resolved by considering how the partial derivative $\partial_{\beta }=\partial /\partial \beta$ differs from the total derivative $d_{\beta }=d/d\beta$. Let us argue about that. On the one hand, the partial derivative of p(t,β,a) with respect to β leads to

(A21) $$\partial_{\beta }\left(p\left(t,\beta ,a\right)\right)=\partial_{\beta }\left(\pi \left(\beta \right)\right)-e^{\lambda (\beta )t}\partial_{\beta }\left(\pi \left(\beta \right)\right)-t\partial_{\beta }\left(\lambda \left(\beta \right)\right)e^{\lambda (\beta )t}\left(\pi \left(\beta \right)-p_{0}\left(a\right)\right)\;,$$

whereby

(A22) $$\partial_{\beta }\left(p(t,\beta ,a)\right){|}_{a=\pi_{2}\left(\beta \right)}=\partial_{\beta }\left(\pi \left(\beta \right)\right)-e^{\lambda (\beta )t}\partial_{\beta }\left(\pi \left(\beta \right)\right)\;.$$

On the other hand, instead, the total derivative is the correct operation that leads to the equality

(A23) $$d_{\beta }\left(p\left(t,\beta ,a\left(\beta \right)=\pi_{2}\left(\beta \right)\right)\right)=\partial_{\beta }\left(p\left(t,\beta ,\pi_{2}\left(\beta \right)\right)\right)=\partial_{\beta }\left(\pi \left(\beta \right)\right)\;.$$

In fact, it holds that

(A24) $$\begin{array}{cc} \;d_{\beta }\left(p\left(t,\beta ,a\left(\beta \right)=\pi_{2}\left(\beta \right)\right)\right) & =\partial_{\beta }\left(p(t,\beta ,a)\right){|}_{a=\pi_{2}\left(\beta \right)}+\partial_{a}\left(p(t,\beta ,a(\beta ))\right)\;d_{\beta }a\left(\beta \right)\\ \; & =\partial_{\beta }\left(\pi \left(\beta \right)\right)-e^{\lambda (\beta )t}\partial_{\beta }\left(\pi \left(\beta \right)\right)+\partial_{a}\left(e^{\lambda (\beta )t}\left[\begin{array}{c} 1-a\\ a \end{array}\right]\right)\partial_{\beta }\left(\pi_{2}\left(\beta \right)\right)\\ \; & =\partial_{\beta }\left(\pi \left(\beta \right)\right)-e^{\lambda (\beta )t}\partial_{\beta }\left(\pi \left(\beta \right)\right)+e^{\lambda (\beta )t}\partial_{\beta }\left(\pi_{2}\left(\beta \right)\right)\left[\begin{array}{c} -1\\ 1 \end{array}\right]\\ \; & =\partial_{\beta }\left(\pi \left(\beta \right)\right)-e^{\lambda (\beta )t}\partial_{\beta }\left(\pi \left(\beta \right)\right)+e^{\lambda (\beta )t}\partial_{\beta }\left(\pi \left(\beta \right)\right) \end{array}$$

(A25) $$\begin{array}{cc} \; & =\partial_{\beta }\left(\pi \left(\beta \right)\right) \end{array}$$

with $\partial_{a}\equiv \partial /\partial a$.

## Appendix C. Derivation of Equation (25)

In this Appendix, we show the derivation of

(A26) $$\partial_{\tilde{\beta }}\left(\rho_{\tilde{\beta }}\right)=\sum_{j=1}^{N}\partial_{\tilde{\beta }}\left(\pi_{j}\left(\tilde{\beta }\right)\right)|\epsilon_{j}\rangle \;\langle \epsilon_{j}|\;,$$

where $\tilde{\beta }$ is the inverse temperature of an initial thermal state, which should not be confused with the inverse temperature β of the thermal bath. As explained in the main text, the computation of Equation (A26) is required to achieve the analytical expression of the QFI of the thermal state $\rho_{\tilde{\beta }}$ in (24). We recall that the latter is given by $F\left(\rho_{\tilde{\beta }},\tilde{\beta }\right)=\mathrm{Var}\left(H\right)$, where $\mathrm{Var}{\left(H\right)}_{\rho_{\tilde{\beta }}}$ is the variance of *H* computed over the thermal state $\rho_{\tilde{\beta }}$.

To derive $\partial_{\tilde{\beta }}\left(\rho_{\tilde{\beta }}\right)$, we compute the derivative of the thermal probabilities $\pi_{j}\left(\tilde{\beta }\right)=e^{-\tilde{\beta }\epsilon_{j}}/Z_{\tilde{\beta }}$ with respect to $\tilde{\beta }$. We recall that $Z_{\tilde{\beta }}=\sum_{j=1}^{N}e^{-\tilde{\beta }\epsilon_{j}}$. Thus,

(A27) $$\partial_{\tilde{\beta }}\left(\pi_{j}\left(\tilde{\beta }\right)\right)=\frac{1}{Z^{2}\left(\tilde{\beta }\right)}\left(\partial_{\tilde{\beta }}\left(e^{-\tilde{\beta }\epsilon_{j}}\right)Z\left(\tilde{\beta }\right)-e^{-\tilde{\beta }\epsilon_{j}}\partial_{\tilde{\beta }}\left(Z\left(\tilde{\beta }\right)\right)\right).$$

Then, we determine $\partial_{\tilde{\beta }}\left(Z\left(\beta \right)\right)$. To do this, it is worth writing the explicit expression of the average of *H* with respect to $\rho_{\tilde{\beta }}$:

(A28) $${\langle H\rangle }_{\rho_{\tilde{\beta }}}=\mathrm{Tr}\left[\rho_{\tilde{\beta }}H\right]=\sum_{j=1}^{N}\epsilon_{j}\pi_{j}\left(\tilde{\beta }\right)=\frac{1}{Z(\tilde{\beta })}\sum_{j=1}^{N}\epsilon_{j}e^{-\tilde{\beta }\epsilon_{j}}=-\frac{1}{Z(\tilde{\beta })}\partial_{\tilde{\beta }}Z\left(\tilde{\beta }\right)\;.$$

Therefore,

(A29) $$\partial_{\tilde{\beta }}Z\left(\tilde{\beta }\right)=-{\langle H\rangle }_{\rho_{\tilde{\beta }}}Z\left(\tilde{\beta }\right).$$

As a result, substituting (A29) into (A27),

(A30) $$\partial_{\tilde{\beta }}\left(\pi_{j}\left(\tilde{\beta }\right)\right)=\left({\langle H\rangle }_{\rho_{\tilde{\beta }}}-\epsilon_{j}\right)\pi_{j}\left(\tilde{\beta }\right),$$

so that

(A31) $$\partial_{\tilde{\beta }}\rho_{\tilde{\beta }}=\sum_{j=1}^{N}\left({\langle H\rangle }_{\rho_{\tilde{\beta }}}-\epsilon_{j}\right)\pi_{j}\left(\tilde{\beta }\right)|\epsilon_{j}\rangle \;\langle \epsilon_{j}|\;.$$
